# Entropy Distribution in a Quantum Informational Circuit of Tunable Szilard Engines

**DOI:** 10.3390/e21100980

**Published:** 2019-10-08

**Authors:** Jose Diazdelacruz

**Affiliations:** Department of Applied Physics and Materials Engineering, Universidad Politecnica de Madrid, 28040 Madrid, Spain; jose.diazdelacruz@upm.es; Tel.: +34-910-676-998

**Keywords:** Szilard engines, quantum thermodynamics, information heat engines

## Abstract

This paper explores the possibility of extending the existing model of a single-particle Quantum Szilard Engine to take advantage of some features of quantum information for driving typical mechanical systems. It focuses on devices that output mechanical work, extracting energy from a single thermal reservoir at the cost of increasing the entropy of a qubit; the reverse process is also considered. In this alternative, several engines may share the information carried by the same qubit, although its interception will prove completely worthless for any illegitimate user. To this end, multi-partite quantum entanglement is employed. Besides, some changes in the cycle of the standard single-particle Quantum Szilard Engine are described, which lend more flexibility to meeting additional requirements in typical mechanical systems. The modifications allow having qubit input and output states of adjustable entropy. This feature enables the possibility of chaining the qubit between engines so that its output state from one can be used as an input state for another. Finally, another tweak is presented that allows for tuning the average output force of the engine.

## 1. Introduction

The capacity of performing mechanical, electrical, or any other kind of work is a valuable resource that is ruled by the laws of Thermodynamics. The second principle precludes the possibility of a device that could cyclically produce work from a single thermal reservoir without interchanging something else with the environment. Information Heat Engines do the job by dumping entropy to the outside. Bits, or their quantum counterparts, qubits, may have different values of entropy. Accordingly, low entropy bits or qubits can be regularly supplied to serve as fuel for Information Heat Engines. They should be returned to a state with larger entropy. Therefore, bits or qubits in a non-maximum entropy state are valuable resources that should be kept hidden from illegitimate users. Under this denomination, we include any unwanted owner of an information heat engine that could intercept a qubit and obtain work in exchange for an entropy increase. Our system avoids this threat by making the reduced state of traveling qubits to be maximally mixed.

Quantum entanglement can be employed to make individual qubits show maximum entropy for anyone except those who have other specially prepared qubits. In other words, adequately entangled qubits are worthless for any possible illegitimate user. This feature makes qubits more adequate than classical bits for being chosen as resources for protected distribution. We describe a system that enables the use of a qubit as a resource for several information heat engines in a row, keeping it protected in its journey from one to the next. Multi-partite GHZ states are used to furnish the required security. The process is described in Section 4 and can be regarded as highly independent from the rest of the paper, where the operation of the individual engines is presented.

A second issue, raised by sharing a qubit between several engines, is the necessity to use qubits with configurable values of input and output entropies. This paper builds on the Quantum Szilard Engine [1] in the single-particle regime. We will refer to it as the reference Quantum Szilard Engine (RefQSZ). This work describes some slight changes that allow to input non-minimum and output non-maximum entropy bits in a way that allows them to be used by several devices consecutively. With this tweak, there is a parallel between hydraulic cylinders and Szilard Engines. In the former, the power that can be output equals the pressure difference times the flow rate, whereas in the latter it equals the baud rate times a constant factor $k_{B}\left(\ln2\right)T$, where $k_{B}$ is Boltzmann’s constant and *T* is the temperature of the available heat reservoir. In order to cope with varying values of the entropy for input and output qubits, a modified cycle is described in Section 5. Two versions of it are presented: one for producing work (motor mode) and another for decreasing the entropy of the circulating qubits (generator mode). More appropriately, from a thermodynamical point of view, the motor/generator denomination should be replaced by motor/refrigerator. However, we chose the former in order to focus on the mechanical energy transfer from/to the device. The process undergone by the qubits is described in Section 6 and the average work balance is analyzed in Section 7.

A comparison with standard hydraulic systems prompts us to address the possibility of delivering the work with tunable values of mechanical force. We will show that the force exerted by the engine on the load can be made arbitrarily high, as it happens with a hydraulic press. Instead of changing the cross-sectional area, just an informational modification of settings is enough in our engine. It is considered in Section 8.

According to the previous paragraphs, improvements over the existing RefQSZ model can be concisely summarized into (a) the use of entanglement, (b) the possibility of varying degrees of entropy in the input and output qubits, and (c) the adaptability of mechanical forces. Finally, when the temperatures of the heat reservoirs of two cylinders differ, they can function according to an equivalent Carnot cycle, that works with just an informational link between the hot and cold engines. We assume that all the processes are slow enough for thermal equilibrium to be maintained all the time. Thus, discussion about any compromise between efficiency and power is avoided.

The paper is organized as follows. Section 2 refers previous papers on the subject. A very succinct description of the RefQSZ is presented in Section 3. The use of GHZ states and the analysis of the changes in entropy are treated in Section 4. A few changes to account for functioning with selectable values of entropies and in motor/generator modes are described in Section 5. The evolution of quantum states and the work output of the engines are analyzed in Section 6 and Section 7. Further tweaks are introduced in Section 8 to tune the output force. Working according to an equivalent Carnot cycle between different temperature reservoirs is considered in Section 9. After that, Section 10 and Section 11 contain some discussions and conclusions, respectively. The partition function and other relevant expressions for a single particle in a cylindrical box are presented in Appendix A. The emergence of a left–right location qubit is described in Appendix B. Feedback mechanisms play an important role in information heat engines. When the path of entropy needs to be carefully accounted for, a neat model of measurement is convenient. In Appendix C, it is reduced to a Controlled-Not (henceforth referred to as CNOT) gate controlled by the qubit to be measured and targeted at another. The ensuing action is then controlled by the target qubit. The initial entropy of the target qubit conditions the fidelity of the measurement and the result of the whole process.

## 2. Antecedents

Steam engines have played a paramount role in the wealth of modern societies since their introduction in the late eighteenth century. They were able to convert the thermal energy resulting from burning coal into mechanical work done on a cylinder rod, which was used in a variety of applications. In the early times of thermodynamics, the French engineer Sadi Carnot [2] set out to study the efficiency of steam engines and came to the remarkable conclusion that there are limitations to the conversion of heat into mechanical work. Even though they are two forms of energy, which is a conserved quantity, further studies also found that heat could not be completely converted into work. This idea was built into the celebrated Second Principle of Thermodynamics and is directly reflected in its formulation by Lord Kelvin [3]. Later on, J. Clerk Maxwell, in his *Theory of Heat* [4] revealed the connection between heat and the kinetic movement of molecules. He posed a long-lasting challenge to the second law by presenting his celebrated demon, which could obtain mechanical work after identifying the velocity of a single molecule.

In 1929 L. Szilard [5] devised an engine made of a cylinder, a piston, and a rod. Inside the cylinder, there was a single gas particle. Knowing which side of the piston the particle was on, allowed the extraction of an amount of work given by $W=k_{B}\left(\ln2\right)T$. The contradiction between Maxwell’s demon or Szilard Engines with the Second Law of Thermodynamics has been finally ruled out by relatively recent papers [1,6,7,8]. They took advantage of the Information Theory put forth by Claude Shannon [9] in 1948. The clue for the solution of the puzzle was, in our view, the link between information and thermodynamics, which underlies the concept of entropy. Knowledge about which side of the piston the molecule is on is equivalent to a reduction of entropy and can only happen cyclically if an information resetting stage takes place, as in computer memories. In 1951, Landauer [10] established that resetting leads to heat dissipation in a thermal reservoir and a work per bit that is equal to the one obtained in Szilard’s engine. This work was measured by [11] in 2012. An updated review on the Thermodynamics of Information can be found in Reference [12,13]. Microscopically, Landauer’s work can be traced to correlation creation rates in collision dynamics [14]. Other devices, like quantum sensors [15], also need resetting steps. The converse of Landauer’s principle also holds. Randomizing previously reset bits may enable the transformation of heat into work, even in the presence of a single temperature reservoir. Bits with preset values become resources for the obtention of work. The stores of bits are known as information reservoirs [16,17,18,19,20], a term coined in Reference [13]. Some papers describe how a string of bits can be processed by information ratchets that interact with simple physical systems with [21] or without thermal reservoirs [22]. There are differences between classical and quantum which have been discussed in several papers [23,24,25,26,27,28], although the Carnot efficiency bound is the same for both [29,30] in the limit of infinitely slow processes and thermal reservoirs. The devices that perform this transformation are known as information heat engines [1,7,31,32,33,34,35,36,37,38,39,40,41,42,43,44,45,46,47]. Moreover, systems of several engines acting collectively may outperform sets of individual stand-alone machines through a suitable transfer of coherence [48].

When a set of bits is used to fuel an information heat engine, there is an upper bound on the work that can be obtained. It is given by the constant $k_{B}\left(\ln2\right)T$ times the Kullback–Leibler divergence (classical bits) or its relative entropy (quantum bits) with respect to their thermal equilibrium states [49,50,51,52]. Assuming degenerate Hamiltonians for the bits, they reduce to standard entropies. Naturally, the magnitude that quantifies this capacity is $N:=S_{\max}-S$, where *S* is the actual value of the entropy and $S_{\max}$ is its maximum. For the classical and quantum bits considered in this paper $S_{\max}=1$ and *N* is known as negentropy [53,54]. Quantum systems with reservoirs at different temperatures, like Otto and Carnot cycles [55,56,57,58] have also been studied.

After realizing that qubits that are not in a maximal entropy state are a valuable resource, it is pertinent to study how to protect them from illegitimate users. It is well known that quantum mechanics [59,60] provides secure procedures that shield communications against potential eavesdropping. Some of them employ entangled states. There have been recent publications [61,62,63] considering correlated or entangled information reservoirs. However, to the best of our knowledge, adapting these techniques to discourage illegitimate users in the context of information heat engines has only been described in our previous papers [64,65]. Both consider bi-partite entanglement between a provider and a consumer of information. In the first, a general scenario is described, while, in the second, we focused on remote electric voltage transformers. These are devices that use the information to move electrons between terminals at different voltages. The present manuscript broadens the scope by using multi-partite entanglement in a way that enables multiple engines to share the same qubit.

## 3. Reference Quantum Szilard Engine

**Figure 1:**
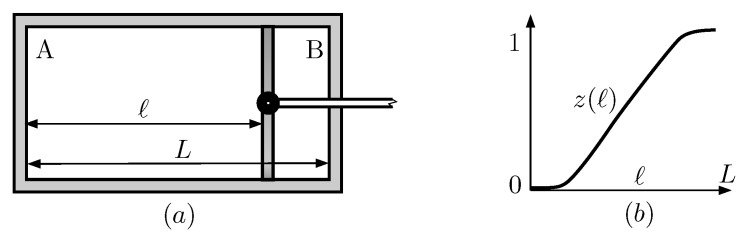
Subfigure (**a**) shows a basic RefQSZ. The length of the cylinder is *L* and the position of the wall is specified by *ℓ*. Subfigure (**b**) represents the function z(ℓ), defined in Equation (7), that denotes the probability for the particle to end in the left compartment when the wall is removed and slowly reintroduced at position *ℓ*. The saturation elbows are quantum effects that are explained physically in References [66,67].

There are different implementations of Maxwell demons and Szilard Engines, both theoretical and experimental. In this work, we use a variation of the RefQSZ described in Reference [1], particularized for the case of a single particle. It features a hollow cylinder of length *L* that is hermetically divided into two compartments by a moving wall, whose position is determined by the distance *ℓ* from the left base. The compartments will be referred to as left (A) or right (B) and their lengths are *ℓ*, L−ℓ, respectively (see Figure 1). In short, on every cycle, a left-right measurement is performed, followed by a full expansion of the piston and a net work of $k_{B}\left(\ln2\right)T$ is obtained. It works cyclically and is continuously in thermal equilibrium with a single reservoir at temperature *T*. Every sequence includes the following four stages:

(I) Insertion of the wall in the middle of the box.
(II) Measurement of which side of the wall the particle is on.
(III) Expansion of the wall until ℓ=L if the particle is on the left side, or until ℓ=0, otherwise.
(IV) Removal of the wall.

**Figure 2:**
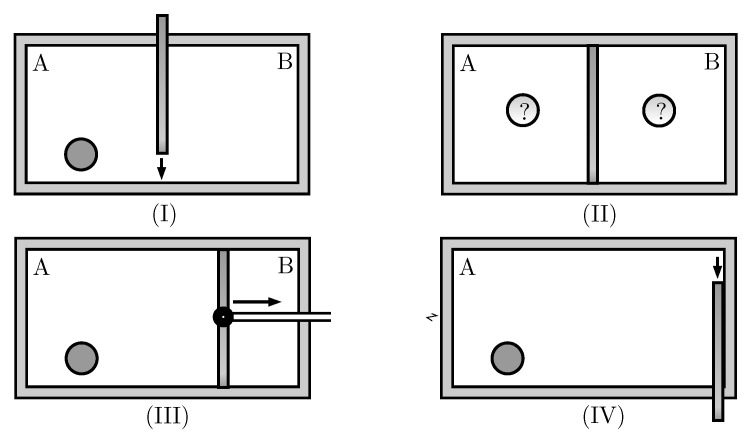
The drawings depict stages (**I**) to (**IV**) of the Quantum Szilard Engine described in Reference [1] for the particular case of only one particle in the cylinder.

They are schematically shown in Figure 2. The bit *M* that hosts the result of the measurement needs either to be replaced by a new blank one, or to be reset. In the latter case, Landauer’s work has to be supplied, which exactly matches the work output of the engine. In the former case, the RefQSZ functions as an engine fueled by fresh blank qubits. Entropy and energy are pulled from the thermal reservoir. The entropy goes to the dumped *M* bit and the energy is extracted as useful work. The second law of Thermodynamics is thus unquestionably observed.

## 4. Protected Information Transmission with GHZ States

**Figure 3:**
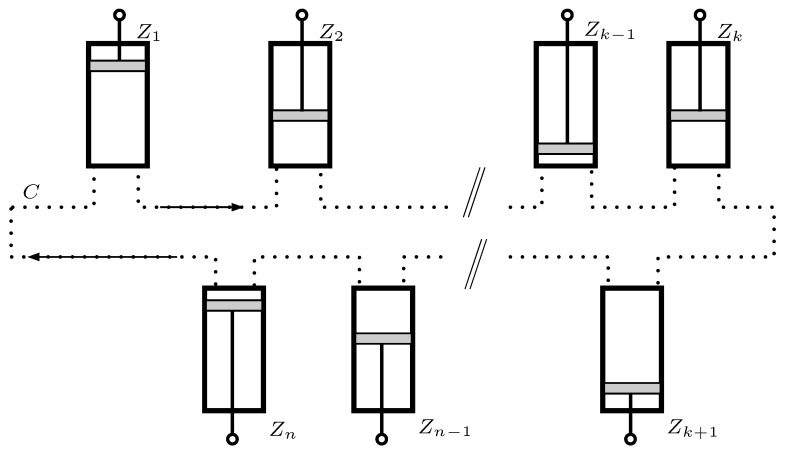
Ring of *n* Szilard cylinders $Z_{1},Z_{2},\ldots ,Z_{n}$ sharing a quantum communication line *C*. The *M* qubits transit the line from a cylinder to the next in a maximally mixed reduced state. Each engine may work either in a motor or a generator mode. In the former, some work is done on the load, whereas in the latter some energy is borrowed.

In this Section, we focus on the distribution of information among a set of *n* Szilard cylinders $Z_{1},\ldots ,Z_{n}$. It is carried by a qubit *M* that moves along a quantum communication line *C* from one engine to the next (see Figure 3). We describe how entanglement can be used to make its reduced state become maximally mixed while it is being transferred. We also show that, upon reception, its previous state is restored. Section 5 shows how each cylinder may increase or decrease the entropy of *M* by producing or consuming mechanical work, respectively. Accordingly, we will consider that Szilard engines may function in either of two modes of operation: motor and generator.

We assume that there is a set of *n* legitimate cylinders $Z_{1},Z_{2},\ldots ,Z_{n}$, which are authorized to use the *M* qubits. They contain a set of local security qubits $G_{1},G_{2},\ldots ,G_{n}$ that are initially in a joint GHZ state $|G\rangle$ defined by

(1) $$|G\rangle =\frac{1}{\sqrt{2}}\;\left(|0,0,\ldots ,0\rangle +|1,1,\ldots ,1\rangle \right).$$

All cylinders share a communication line *C* over which a qubit *M* is sent from one to the next. Prior to placing *M* in *C*, let us assume that it is located within cylinder $Z_{k-1}$ and that its initial state $\rho_{0}^{\left(M\right)}$ is given by

(2) $$\rho_{0}^{\left(M\right)}=\chi_{0}|0\rangle \langle 0|+\frac{1-\chi_{0}}{2}\;U_{M},$$

where $U_{M}$ is the identity operator in the Hilbert space of *M*, and $0\leq \chi_{0}\leq 1$. The endpoints $\chi_{0}=0,1$ represent the maximally mixed, and the pure $|0\rangle$ states, respectively. Before sending *M* from $Z_{k-1}$ to $Z_{k}$, it undergoes a CNOT gate controlled by the $G_{k-1}$ qubit. Thus, a joint entangled state

(3) $$\begin{array}{cc} \rho_{0}^{\left(MG\right)}= & \frac{1}{2}\;\chi_{0}\left(|0,0,0,\ldots ,0\rangle +|1,1,1,\ldots ,1\rangle \right)\left(\langle 0,0,0,\ldots ,0|+\langle 1,1,1,\ldots ,1|\right)\\ & +\frac{1-\chi_{0}}{4}\;\left(|0\rangle \langle 0|+|1\rangle \langle 1|\right)\;⊗\;\left(|0,0,\ldots ,0\rangle +|1,1,\ldots ,1\rangle \right)\left(\langle 0,0,\ldots ,0|+\langle 1,1,\ldots ,1|\right) \end{array}$$

is formed. Its reduced state for *M* is the maximally mixed state $U_{M}/2$. Accordingly, *M* can be sent to $Z_{k}$, so that its interception would prove fruitless for the Szilard engine of any illegitimate user.

Upon reception, $Z_{k}$ applies another CNOT gate on *M* controlled by the local $G_{k}$ qubit. The resulting joint state is

(4) $$\begin{array}{cc} \rho_{1}^{\left(MG\right)}= & \frac{1}{2}\;\chi_{0}\left(|0,0,0,\ldots ,0\rangle +|0,1,1,\ldots ,1\rangle \right)\left(\langle 0,0,0,\ldots ,0|+\langle 0,1,1,\ldots ,1|\right)\\ & +\frac{1-\chi_{0}}{4}\;\left(|0\rangle \langle 0|+|1\rangle \langle 1|\right)\;⊗\;\left(|0,0,\ldots ,0\rangle +|1,1,\ldots ,1\rangle \right)\left(\langle 0,0,\ldots ,0|+\langle 1,1,\ldots ,1|\right), \end{array}$$

where it is possible to factor out the state of the *M* qubit, rewriting Equation (4) as

(5) $$\begin{array}{cc} \rho_{1}^{\left(MG\right)}= & \left(\chi_{0}|0\rangle \langle 0|+\frac{1-\chi_{0}}{2}\;U_{M}\right)\;⊗\\ & \frac{1}{2}\left(|0,0,\ldots ,0\rangle +|1,1,\ldots ,1\rangle \right)\left(\langle 0,0,\ldots ,0|+\langle 1,1,\ldots ,1|\right). \end{array}$$

**Figure 4:**
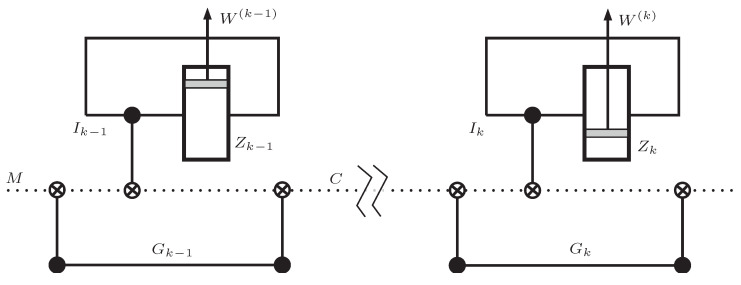
Representation of the *M* qubit processing by consecutive cylinders. First, a CNOT is applied, controlled by the local security qubit $G_{k}$. It unlocks the *M* qubit, so that it can undergo a second CNOT, controlled by the internal qubit *I*. After $Z_{k}$ has finished its cycle, a new CNOT, again controlled by $G_{k}$ prepares *M* for the transmission to $Z_{k+1}$.

The *M* qubit is now available to be used in the local cylinder $Z_{k}$. After it is processed, it can be transmitted to $Z_{k+1}$, provided that it is left in the state represented in Equation (2) with possibly another value of $\chi_{0}$. Figure 4 represents the process.

## 5. Partial Information Cycles for Motors and Generators

In this section, we outline two cycles, one for the motor mode, and the other for the generator mode. Even though they could be viewed as the inverse of each other, we describe both of them explicitly. The reason is that there are subtle differences between the direct and the reverse paths. The first difference with RefQSZ is that we do not assume that the measurement completely determines which side of the wall the particle is on. As we show in Section 6, this is equivalent to assuming that the input state of the *M* qubit is not pure. Another difference with RefQSZ is that the insertion of the wall does not induce equal probabilities for the left and right locations. This will leave the *M* qubit in a partially mixed state.

Next, we describe the modified cycle for the motor mode and compare it with the one presented in Reference [1] for the RefQSZ. The stages are:

(I) Insertion. Instead of reinserting the wall in the middle of the box, we consider the case where it is reintroduced at an arbitrary position, determined by the value $\ell_{1}$ of *ℓ*. It is assumed that $\ell_{1}\geq L/2$. Considering the left-right symmetry of the cylinder, this restriction does not imply a loss of generality. At the end of this stage there is no tunneling between the left and the right compartments.
(II) Measurement. We assume that the fresh qubit *M* which is used to host the result of the measurement enters a partially mixed state. The which-side qubit *I*, defined in Appendix B and determined by the left ($|0\rangle$) or right ($|1\rangle$) situation of the particle, enters the stage in a state
  (6) $$\rho_{0}^{\left(I\right)}=\alpha |0\rangle \langle 0|+\left(1-\alpha \right)|1\rangle \langle 1|,$$
  where $1\geq \alpha >\frac{1}{2}$ and α only depends on $\ell_{1}$. It is the probability of finding the particle on the left side at the end of the insertion process. It can be computed from the partition function $Z_{0}\left(\ell \right)$ for the particle in a cylindrical box given in Appendix A. Precisely, $\alpha =z(\ell_{1})$, where
  (7) $$z\left(\ell \right):=\frac{Z_{0}\left(\ell \right)}{Z_{0}\left(\ell \right)+Z_{0}\left(L-\ell \right)}.$$
  In the original RefQSZ, the measurement was perfect, which is equivalent to having a pure $\chi_{0}=1$ initial state for *M*; it also assumed that $\ell_{1}=L/2$, which implied α=1/2.
(III) Expansion. Considering that the left/right measurement is not completely certain, the expansion will stop at a suitable value $\ell_{2}^{\left(M=0,1\right)}$ of *ℓ*, controlled by the result M=0,1 of the measurement.
(IV) Removal. This stage differs from the RQuSZE only in that the wall is pulled-out at $\ell =\ell_{2}^{\left(M=0,1\right)}$, instead of at ℓ=L or ℓ=0. The positions $\ell_{2}^{\left(M=0,1\right)}$ are chosen so that the extraction is reversible. Therefore, they are determined by the equations:
  (8) $$\left\{\begin{array}{ccc} z(\ell_{2}^{\left(M=0\right)}) & = & r_{00}\\ z(\ell_{2}^{\left(M=1\right)}) & = & r_{01} \end{array}\right.,$$
  where $r_{00},r_{01}$ are the conditional probabilities for the particle to be on the left side for M=0,1, respectively. They are derived in Section 6 and written in Equation (12).

The cycle is intended to obtain some average value of work in three isothermal and reversible stages (I, III and IV) and to pump out some entropy at stage (II). The obtention of the average value of work is presented in Section 7.

By reversing the previous stages, it is possible to tweak the engine so that it operates as a generator. In this mode, the entropy of the *M* qubit decreases at the cost of absorbing some work. The steps of the cycle are:

(I’) Insertion. The position of the insertion $\ell_{1}^{\left(M=0,1^{\prime }\right)}$ is controlled by *M*. This creates a correlation between *M* and the left/right position of the particle.
(II’) Compression. The wall is displaced from its initial position to a final value of $\ell_{2}^{\prime }$.
(III’) Erasure. A CNOT gate, controlled by the position of the particle, is applied to *M*. As a consequence, some of the entropy of *M* is transferred to *I* and their joint state factorizes.
(IV’) Removal. The wall is pulled-out at $\ell =\ell_{2}^{\prime }$.

**Figure 5:**
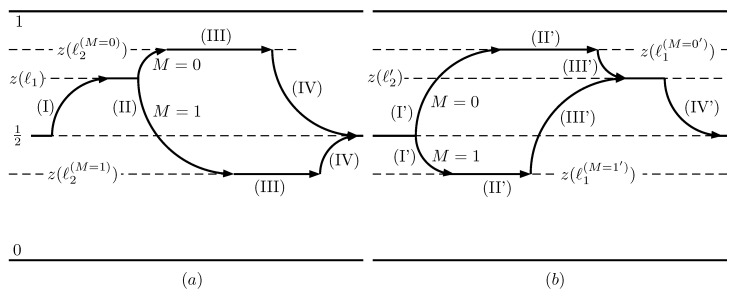
On the left, subfigure (**a**) depicts the stages of the motor cycle. The vertical axis represents the conditional probability of finding the particle on the left side of the wall. The right subfigure (**b**) corresponds to the generator mode. Function z(ℓ) is defined in Equation (7).

Interestingly, the reverse of the measuring stage is the erasure. The former creates a correlation between *I* and *M*, while the latter makes the state of *M* completely independent of *I*. Figure 5 represents the cycles of the two modes.

## 6. Qubit Processing by A Cylinder

In this Section, we describe the evolution of the quantum state of the system defined by the *M* and *I* qubits along the different stages of the cycles. Following Section 4, once the *M* qubit reaches cylinder $C_{k}$, it becomes the target of a CNOT gate controlled by the local qubit $G_{k}$. The *M* qubit is now in the state described by Equation (4) and enters either a motor or a generator cycle. We analyze them separately.

(a) Motor. In the first stage, the introduction of the wall at $\ell =\ell_{1}$ sets the state of *I* to be partially mixed and is given by Equation (6).
  Next, in stage (II), the *M* qubit is used as target in a CNOT gate controlled by the which-side qubit *I*. After the CNOT gate, the joint state of qubits *I*,*M* reads
  (9) $$\begin{array}{cc} \rho_{1}^{\left(IM\right)}= & \alpha \frac{1+\chi_{0}}{2}|00\rangle \langle 00|+\left(1-\alpha \right)\frac{1-\chi_{0}}{2}|10\rangle \langle 10|+\\ & \alpha \frac{1-\chi_{0}}{2}|01\rangle \langle 01|+\left(1-\alpha \right)\frac{1+\chi_{0}}{2}|11\rangle \langle 11|. \end{array}$$
  The *M* qubit then controls the final position in the expansion stage. Its two states $\left\{|0\rangle ,|1\rangle \right\}$ have probabilities $p_{0},p_{1}$, that are given by
  (10) $$\left\{\begin{array}{ccc} p_{0} & = & \frac{1-\chi_{0}+2\alpha \chi_{0}}{2}\\ p_{1} & = & \frac{1-\chi_{0}+2\left(1-\alpha \right)\chi_{0}}{2} \end{array}\right..$$
  The states of *I*, conditioned on *M* are:
  (11) $$\left\{\begin{array}{ccc} \rho_{M=0}^{I} & = & r_{00}|0\rangle \langle 0|+r_{10}|1\rangle \langle 1|\\ \rho_{M=1}^{I} & = & r_{01}|0\rangle \langle 0|+r_{11}|1\rangle \langle 1| \end{array}\right.,$$
  where
  (12) $$\left\{\begin{array}{ccc} r_{00} & = & \frac{\alpha (1+\chi_{0})}{1-\chi_{0}+2\alpha \chi_{0}}\\ r_{10} & = & \frac{\left(1-\alpha \right)\left(1-\chi_{0}\right)}{1-\chi_{0}+2\alpha \chi_{0}}\\ r_{01} & = & \frac{\alpha (1-\chi_{0})}{1-\chi_{0}+2\left(1-\alpha \right)\chi_{0}}\\ r_{11} & = & \frac{\left(1-\alpha \right)\left(1-\chi_{0}\right)}{1-\chi_{0}+2\left(1-\alpha \right)\chi_{0}} \end{array}\right.,$$
  are the conditional probabilities of *I* given *M*, in the computational basis.
  The *M* qubit leaves the cycle in the state
  (13) $$\rho_{1}^{\left(M\right)}=p_{0}|0\rangle \langle 0|+p_{1}|1\rangle \langle 1|,$$
  which, after defining
  (14) $$\chi_{1}:=2p_{0}-1,$$
  becomes
  (15) $$\rho_{1}^{\left(M\right)}=\chi_{1}|0\rangle \langle 0|+\frac{1-\chi_{1}}{2}\;U,$$
  which leads to the relation between $\chi_{0},\chi_{1}$ as a function of α
  (16) $$\chi_{1}=\chi_{0}\;\left(2\alpha -1\right).$$
(b) Generator. This mode is essentially the inverse of the motor mode. However, for the sake of disambiguation, we outline it in detail. The objective of the process is to output the *M* qubit in state
  (17) $$\rho_{1}^{\left(M\right)}=\chi_{1}^{\prime }\;|0\rangle \langle 0|+\frac{1-\chi_{1}^{\prime }}{2}\;U_{M},$$
  when its input state is
  (18) $$\rho_{0}^{\left(M\right)}=\chi_{0}^{\prime }|0\rangle \langle 0|+\frac{1-\chi_{0}^{\prime }}{2}\;U_{M},$$
  assuming $0\leq \chi_{0}^{\prime }<\chi_{1}^{\prime }<1$. With the benefit of hindsight, after the description of the motor mode, we determine a set of relevant parameters for the generator mode. Looking at Equation (16), we define
  (19) $$\alpha^{\prime }=\frac{1}{2}+\frac{\chi_{0}^{\prime }}{2\chi_{1}^{\prime }},$$
  which always lies in the interval $\left[\frac{1}{2},1\right]$. From Equation (14) we define
  (20) $$p_{0}^{\prime }=\frac{1+\chi_{0}^{\prime }}{2}\;,\;p_{1}^{\prime }=1-p_{0}^{\prime },$$
  and from Equation (11),
  (21) $$\left\{\begin{array}{ccc} \rho_{M=0}^{I^{\prime }} & = & \frac{1}{2p_{0}^{\prime }}\left(\alpha^{\prime }\left(1+\chi_{1}^{\prime }\right)|0\rangle \langle 0|+\left(1-\alpha^{\prime }\right)\left(1-\chi_{1}^{\prime }\right)|1\rangle \langle 1|\right)\\ \rho_{M=1}^{I^{\prime }} & = & \frac{1}{2p_{1}^{\prime }}\left(\alpha \left(1-\chi_{1}^{\prime }\right)|0\rangle \langle 0|+\left(1-\alpha \right)\left(1+\chi_{1}^{\prime }\right)|1\rangle \langle 1|\right) \end{array}\right..$$
  States $\rho_{M=0}^{I}{\;}^{\prime },\rho_{M=1}^{I}{\;}^{\prime }$ determine $\ell_{2}^{\left(M=0\right)}{\;}^{\prime },\ell_{1}^{\left(M=1\right)}{\;}^{\prime }$ as the positions for a reversible reintroduction of the wall, so that it is controlled in Stage (I’) by qubit *M*. Accordingly, with probabilities $p_{0}^{\prime }/p_{1}^{\prime }$, the *I* qubit exits (I’) in states $\rho_{M=0}^{I^{\prime }}/\rho_{M=1}^{I^{\prime }}$, respectively.
  The next stage is still controlled by *M* and leads to a position of the wall given by $\ell_{2}^{\prime }$. It is determined by the condition that if the wall was reinserted at $\ell_{2}^{\prime }$, the reduced state of *I* would be
  (22) $$\rho_{1}^{\left(I^{\prime }\right)}=\;\alpha^{\prime }\;|0\rangle \langle 0|\;+\;\left(1-\alpha^{\prime }\right)\;|1\rangle \langle 1|,$$
  Then, after the CNOT on *M* controlled by *I*, the joint state factorizes into
  (23) $$\rho_{1}^{\left(IM^{\prime }\right)}=\rho_{1}^{\left(I^{\prime }\right)}⊗\left[\frac{1+\chi_{1}^{\prime }}{2}|0\rangle \langle 0|+\frac{1-\chi_{1}^{\prime }}{2}|1\rangle \langle 1|\right],$$
  which is ready for a reversible wall removal at $\ell_{2}^{\prime }$.

## 7. Work Evaluation

This Section considers the work output/input of the motor/generator modes. As a first observation, we must point out that it is a random variable and we are only interested in its average value. The wall does not move in the stages (II) and (III’). Therefore, the work vanishes in both steps. Accordingly, we only consider the remaining stages, assuming that they proceed reversibly and in thermal equilibrium at temperature *T*. These assumptions allow us to compute the average work $<W^{\left(X\right)}>$ in stage (X) as the decrement of the free energy *F* for the particle in the box

(24) $$<W^{\left(X\right)}>=F^{\left(X_{i}\right)}-F^{\left(X_{f}\right)},$$

being $F^{\left(X_{i}\right)}/F^{\left(X_{f}\right)}$ the free energies at the beginning/end of stage (X). The free energy for the state $\rho^{\left(P\right)}$ of the particle in the cylinder can be computed as

(25) $$F_{\rho^{\left(P\right)}}=\mathrm{Tr}\;\left\{H\rho^{\left(P\right)}\right\}-k_{B}\left(\ln2\right)TS_{\rho^{\left(P\right)}},$$

where *H* is the Hamiltonian and $S_{\rho^{\left(P\right)}}$ is the entropy of $\rho^{\left(P\right)}$. Next, we consider separately the motor and generator modes.

(a) Motor. After addition of the (I), (III) and (IV) contributions, we arrive at
  (26) $$<W^{\left(I\right)}+W^{\left(III\right)}+W^{\left(IV\right)}>=F^{\left(II_{f}\right)}-F^{\left(II_{i}\right)}.$$
  Following the measurement, there are two possible states for the particle, $\rho_{M=0}^{\left(P\right)},\rho_{M=1}^{\left(P\right)}$, with probabilities $p_{0},p_{1}$, respectively. The average work expected when M=0 is
  (27) $$<W{>}_{M=0}=\mathrm{Tr}\;\left\{(\rho_{M=0}^{\left(P\right)}-\rho_{0}^{\left(P\right)})H\right\}+k_{B}\left(\ln2\right)T\left(S_{0}^{\left(P\right)}-S_{M=0}^{\left(P\right)}\right),$$
  while if M=1, it is
  (28) $$<W{>}_{M=1}=\mathrm{Tr}\;\left\{(\rho_{M=1}^{\left(P\right)}-\rho_{0}^{\left(P\right)})H\right\}+k_{B}\left(\ln2\right)T\left(S_{0}^{\left(P\right)}-S_{M=1}^{\left(P\right)}\right),$$
  where $S_{0}^{\left(P\right)}$ is the entropy of the *P* state before the CNOT, and $S_{M=0}^{\left(P\right)},S_{M=1}^{\left(P\right)}$ conditioned to M=0,1, after the CNOT gate.
  Consequently, the average value of the work per cycle yields
  (29) $$\begin{array}{cc} <W>= & p_{0}<W{>}_{0}+p_{1}<W{>}_{1}\\ = & \mathrm{Tr}\;\left\{(p_{0}\;\rho_{M=0}^{\left(P\right)}+p_{1}\;\rho_{M=1}^{\left(P\right)}-\rho_{0}^{\left(P\right)})H\right\}+k_{B}\left(\ln2\right)T\left(S_{0}^{\left(P\right)}-p_{0}S_{M=0}^{\left(P\right)}-p_{1}S_{M=1}^{\left(P\right)}\right), \end{array}$$
  where the first contribution cancels, because $\rho_{0}^{\left(P\right)}=p_{0}\;\rho_{M=0}^{\left(P\right)}+p_{1}\;\rho_{M=1}^{\left(P\right)}$. Thus,
  (30) $$<W>=k_{B}\left(\ln2\right)T\left(S_{0}^{\left(P\right)}-p_{0}S_{M=0}^{\left(P\right)}-p_{1}S_{M=1}^{\left(P\right)}\right).$$
  We can derive another equation by considering that the CNOT gate preserves the entropy of the *M*-*P* system. Before the CNOT, *M*,*P* are independent. Therefore,
  (31) $$S_{0}^{\left(MP\right)}=S_{0}^{\left(P\right)}+S_{0}^{\left(M\right)}.$$
  On the other hand, after the CNOT, the two possible states of *P*: $\rho_{M=0}^{\left(P\right)},\rho_{M=0}^{\left(P\right)}$ occur with probabilities $p_{0},p_{1}$. Then,
  (32) $$S_{1}^{\left(MP\right)}=g\left(p_{0}\right)+p_{0}S_{M=0}^{\left(P\right)}+p_{1}S_{M=1}^{\left(P\right)},$$
  where $S_{1}^{\left(MP\right)}$ is the entropy of the *M*-*P* system after the CNOT, and g(x):=−xlogx−(1−x)log(1−x) is the well-known Shannon entropy function. After equating Equations (31) and (32), and substituting $g(p_{0})$ for $S_{1}^{\left(M\right)}$, we arrive at:
  (33) $$S_{0}^{\left(P\right)}+S_{0}^{\left(M\right)}=S_{1}^{\left(M\right)}+p_{0}S_{M=0}^{\left(P\right)}+p_{1}S_{M=1}^{\left(P\right)},$$
  which, combined with Equation (30), yields
  (34) $$<W_{c}>=k_{B}\left(\ln2\right)T\;\left[S_{1}^{\left(M\right)}-S_{0}^{\left(M\right)}\right]=k_{B}\left(\ln2\right)T\;\Delta S^{\left(M\right)},$$
  where $\Delta S^{\left(M\right)}$ represents the entropy increment of *M* in the cycle.
  As has been shown, the CNOT gate plays a central role in the obtention of the average work per cycle. The following remarks may shed some additional intuition on the previous derivation. The only contribution to the energy of the system, before and after the CNOT, comes from the particle, because the *M* qubit is assumed to have a completely degenerate Hamiltonian. Accordingly, only the reduced state of the particle is relevant. Note that it is not changed by the CNOT gate. However, this does not imply that the energies of the conditional states $\rho_{M=0,1}^{\left(P\right)}$ are equal. Considering the entropy, the distinctive character of the CNOT stems from the fact that it is the only interaction that exchanges information between the particle and the *M* qubits. Despite the overall entropy being conserved by the gate, the conditional states $\rho_{M=0,1}^{\left(P\right)}$ and $\rho_{0}^{\left(P\right)}$ show different values and the entropy of *M* is raised. This increment is the prize paid for the obtained work.
(b) Generator. Considering that the generator and motor modes are the reverse of one another, Equation (34) applies. The only obvious difference is that in the generator mode, $<W_{c}>,\Delta S^{\left(M\right)}$ are both negative.

## 8. Force Matching Tweak

The Szilard Engines described so far trade work for bits at a fixed rate of $k_{B}\left(\ln2\right)T$. However, the average force at which they deliver or receive the work can not be arbitrarily chosen. Besides, the insertion and extraction of the wall are not energy neutral. As a consequence, not all the average work is delivered through the piston rod. In this Section, a new tweak is described that works for both motors and generators. It allows for arbitrarily tuning the force on the load and delivering all the average work to it.

From the work calculation of Section 7, it is clear that the average work per cycle is solely determined by the (II) and (III’) stages in the motor and generator cycles, respectively. It does not mean that the work is only done in (II) or (III’) stages. In fact, the average work in them is null. The work on the wall is delivered in the remaining stages. This consideration leads to the following conclusion: designing a new step that accounts for the whole average work of the cycle leaves the remaining stages with a vanishing value. This new step should be fitted into the cycle and proceed in a reversible isothermal way. Its purpose is to allow the selection of the average output force. The load should only be engaged during the new step. In the motor cycle, the following changes are introduced:

1. A new stage (IIa) known as Precompression is defined between (II) and (III). The wall is moved from $\ell_{1}$ to a suitable position $\ell_{3}^{\left(M=0,1\right)}$, controlled by *M* and keeping the load disengaged.
2. Stage (III) now proceeds from $\ell_{3}^{\left(M=0,1\right)}$ to $\ell_{4}^{\left(M=0,1\right)}$. The load is engaged only in this stage, and the values of $\ell_{4}^{\left(M=0,1\right)}$ are also controlled by *M*.
3. A second new stage (IIIa), called **Relaxation**, follows (III). The wall is moved to $\ell_{2}^{\left(M=0,1\right)}$ defined in Section 5, keeping the load disengaged.

The values of $\ell_{3}^{\left(M=0,1\right)},\ell_{4}^{\left(M=0,1\right)}$ are settable. They are determined from two specifications that can be chosen before each cycle. The first one is the average work $<W_{c}>$ expected from the engine, or, equivalently, the entropy variation of *M*, given in Equation (34). The second constraint is the mean value $f_{m}$ in the interval of the expected force. A straightforward equation is obtained:

(35) $$\ell_{4}^{\left(M=0,1\right)}-\ell_{3}^{\left(M=0,1\right)}=k_{B}\left(\ln2\right)T\;\frac{\Delta S^{\left(M\right)}}{f_{m}}$$

The expected force exerted on the piston at position *ℓ* is

(36) $$<f\left(\ell \right)>=k_{B}T<\frac{\partial \;\;(\ln Z)}{\partial \;\;\ell }>,$$

which, conditioned on *M*, is given by

(37) $$<f\left(\ell \right)>=\left\{\begin{array}{ccc} <f^{\left(M=0\right)}\left(\ell \right)>= & k_{B}T\left(r_{00}\frac{\partial \;\;(\ln Z_{0}\left(\ell \right))}{\partial \;\;\ell }-r_{10}\frac{\partial \;\;(\ln Z_{0}\left(L-\ell \right))}{\partial \;\;\ell }\right) & ,\mathrm{for}M=0\\ <f^{\left(M=1\right)}\left(\ell \right)>= & k_{B}T\left(r_{01}\frac{\partial \;\;(\ln Z_{0}\left(\ell \right))}{\partial \;\;\ell }-r_{11}\frac{\partial \;\;(\ln Z_{0}\left(L-\ell \right))}{\partial \;\;\ell }\right) & ,\mathrm{for}M=1 \end{array}\right.,$$

where $r_{ij}$ represent the conditional probabilities for *I*, given *M*. They can be read off directly from Equation (12). The function $Z_{0}\left(\ell \right)$ is given in Appendix A. The mean value $f_{m}$ of <f(ℓ)> over the interval $\left(\ell_{3}^{\left(M=0,1\right)},\ell_{4}^{\left(M=0,1\right)}\right)$ is defined by

(38) $$f_{m}=\frac{1}{\ell_{4}^{\left(M=0,1\right)}-\ell_{3}^{\left(M=0,1\right)}}\;\int_{\ell_{3}^{\left(M=0,1\right)}}^{\ell_{4}^{\left(M=0,1\right)}}<f^{\left(M=0,1\right)}\left(\ell \right)>d\ell ,$$

that, introducing the free energies

(39) $$\left\{\begin{array}{ccc} F^{\left(M=0\right)}\left(\ell \right) & = & -k_{B}T\left(r_{00}\left(\ln Z_{0}\left(\ell \right)\right)+r_{10}\left(\ln Z_{0}\left(L-\ell \right)\right)\right)\\ F^{\left(M=1\right)}\left(\ell \right) & = & -k_{B}T\left(r_{01}\left(\ln Z_{0}\left(\ell \right)\right)+r_{11}\left(\ln Z_{0}\left(L-\ell \right)\right)\right) \end{array}\right.,$$

yields the second equation

(40) $$f_{m}=\frac{1}{\ell_{4}^{\left(M=0,1\right)}-\ell_{3}^{\left(M=0,1\right)}}\;\langle F^{\left(M=0,1\right)}\left(\ell_{3}^{\left(M=0,1\right)}\right)-F^{\left(M=0,1\right)}\left(\ell_{4}^{\left(M=0,1\right)}\right)\rangle ,$$

which, together with Equation (35), yields the values of $\ell_{3}^{\left(M=0,1\right)},\ell_{4}^{\left(M=0,1\right)}$. In Section 10, we discuss the existence of solutions for different values of $f_{m}$ and $<W_{c}>$ (or, equivalently, $\Delta S^{\left(M\right)}$).

For the generator mode, the following changes are introduced:

1. A new stage (I’a) that we also call Precompression is defined between (I’) and (II’). The wall is moved to a suitable position $\ell_{3}^{\left(M=0,1^{\prime }\right)}$, keeping the load (in the generator mode the load acts supplying energy, as a pump in a hydraulic system) disengaged. The value of $\ell_{3}^{\left(M=0,1^{\prime }\right)}$ is controlled by *M*.
2. Stage (II’) now proceeds from $\ell_{3}^{\left(M=0,1^{\prime }\right)}$ to $\ell_{4}^{\left(M=0,1^{\prime }\right)}$. The load is engaged only in this stage and the value of $\ell_{4}^{\left(M=0,1^{\prime }\right)}$ is also controlled by *M*.
3. A second new stage (II’a), also called Relaxation, follows (II’). Te wall is moved to $\ell_{2}^{\prime }$ keeping the load disengaged.

**Figure 6:**
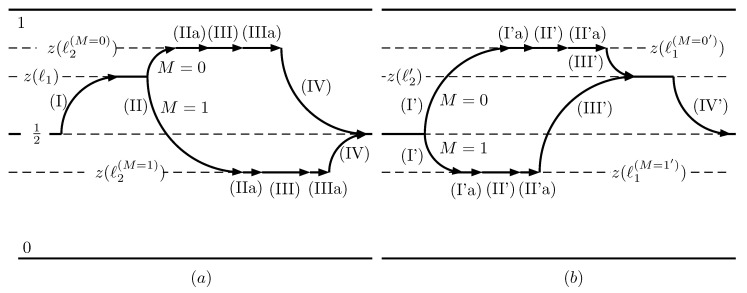
On the left, subfigure (**a**) depicts the stages of the motor cycle with the additional (IIa), (IIIa) stages. Subfigure (**b**) corresponds to the generator mode with the additional (I’a), (II’a) stages. The vertical axis represents the conditional probability of finding the particle on the left side of the wall for the two alternatives M=0,1. Function z(ℓ) is defined in Equation (7).

The selection of $\ell_{3}^{\left(M=0,1^{\prime }\right)},\ell_{4}^{\left(M=0,1^{\prime }\right)}$ follows the same conditions expressed by Equations (35) and (40). Figure 6 represents the cycles of the two modes.

## 9. Carnot Cycles with Szilard Cylinders

The previous sections showed how to obtain work from increasing the entropy of a qubit. The reverse process has also been explained. In both situations, it has been established that the work value of a bit is $k_{B}\left(\ln2\right)T$. If the temperatures of motor and generator are different, then there is a cycle that leads to a net work output. Qubit *M* should be reset at the cold generator, and its entropy increased at the hot motor. After both steps, the qubit is left in its original state, and there is a net work per cycle given by

(41) $$<W>=k_{B}\;\left(\ln2\right)\;\left(T_{h}-T_{c}\right)\;\Delta S,$$

where ΔS is the entropy increase at the motor, which is equal to its decrease at the generator.

The heat drawn from the hot reservoir is $k_{B}\left(\ln2\right)T_{h}$, while the heat released to the cold one is $k_{B}\left(\ln2\right)T_{c}$. The engine efficiency matches that of typical Carnot cycles. The reverse (refrigeration) process is also possible.

## 10. Discussion

The cycles described for the motor and generator modes in Section 6 led to Equation (16), from which it is evident that any positive or negative variation of the entropy of qubit *M* is possible.

Section 4 explained how the *M* qubit could be transmitted between different cylinders so that they were useless while being on their way from one cylinder to the next.

We have left for this section the discussion on the possibility of choosing the average output force by means of the tweaks described in Section 8. In the following, we assume that $0<\chi_{0}<1$.

**Figure 7:**
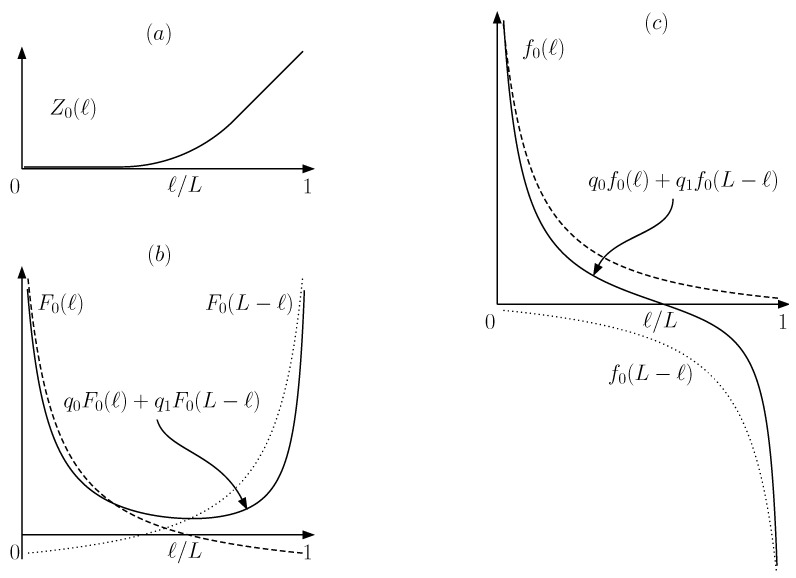
Graphical representations of partition functions (**a**), free energies (**b**) and forces (**c**) as a function of the wall position *ℓ*. Graphs (**b**) and (**c**) represent the case of the particle on the left side (dashed line), on the right side (dotted line) and a linear combination with weights $r_{00},r_{10}$ of both.

We refer to the analytical expressions given in Appendix A for the partition function $Z_{0}\left(\ell \right)$, its associated free energy $F_{0}\left(\ell \right)=-k_{B}T\;\ln Z_{0}\left(\ell \right)$ and force $f_{0}\left(\ell \right)=-\frac{\partial \;\;F_{0}\left(\ell \right)}{\partial \;\;\ell }$. Figure 7 shows graphical representations of $Z_{0}\left(\ell \right),F_{0}\left(\ell \right),F^{\left(M=0,1\right)},f_{0}\left(\ell \right),f^{\left(M=0\right)}\left(\ell \right)$.

In the motor mode, the average output work on the load per cycle of the cylinder is $<W_{c}>$ and is computed taking into account the probabilities for all the possible values of *M* and *I*

(42) $$<W_{c}>=p_{0}W_{c}^{\left(M=0\right)}+p_{1}W_{c}^{\left(M=1\right)},$$

where $W_{c}^{\left(M=0\right)},W_{c}^{\left(M=1\right)}$ are the average work values that correspond to the possible values of *M*. We set $<W_{c}>=W_{c}^{\left(M=0\right)}=W_{c}^{\left(M=1\right)}$. For $M=|0\rangle$, the particle is more likely on the left side of the wall. In this case, the load is attached so that its force on the rod is directed to the left. For $M=|1\rangle$, Equation (11) determines whether the particle is more likely on the left or the right. In the first case, the load is directed to the left, whereas in the second it is directed to the right. We will pick the $M=|0\rangle$ case and leave out the $M=|1\rangle$ alternative because its discussion would be extremely similar. The average work supplied to the load in the expansion stage is

(43) $$W_{c}^{\left(M=0\right)}=r_{00}\left[F_{0}\left(\ell_{3}^{\left(M=0\right)}\right)-F_{0}\left(\ell_{4}^{\left(M=0\right)}\right)\right]+r_{10}\left[F_{0}\left(L-\ell_{3}^{\left(M=0\right)}\right)-F_{0}\left(L-\ell_{4}^{\left(M=0\right)}\right)\right],$$

where $\ell_{3}^{\left(M=0\right)},\ell_{4}^{\left(M=0\right)}$ are chosen following Equations (35) and (40). Alternatively, they are fixed by fitting the given vertical difference $<W_{c}>$ to the given $\ell_{4}^{\left(M=0\right)}-\ell_{3}^{\left(M=0\right)}$ horizontal interval in the graphical representation of $F^{\left(M=0\right)}\left(\ell \right)$ shown by the solid line in Figure 7b. Considering the U-shape and the absence of upper bounds at both endpoints of the domain, any $<W_{c}>$ can be accommodated vertically in infinite ways. Each one is characterized by a different $\ell_{4}^{\left(M=0\right)}-\ell_{3}^{\left(M=0\right)}$ width. Since it can be chosen arbitrarily small, there is no upper bound for $f_{m}$. However, given $\Delta S^{\left(M\right)}$ (or, equivalently, $<W_{c}>$), there is a lower bound

(44) $$f_{m}>k_{B}\left(\ln2\right)T\;\frac{\Delta S^{\left(M\right)}}{L},$$

which stems from the finite size of the cylinder. It can not be reached because of the reasonable restrictions $\ell_{3}^{\left(M=0\right)}>0,\ell_{4}^{\left(M=0\right)}<L$, but can be approached arbitrarily closely from above. Of course, this bound can be reduced by dividing $\Delta S^{\left(M\right)}$, at the expense of doing more cycles for the same amount of work.

The variance of the output work results

(45) $$\sigma_{W}^{2}=r_{00}r_{10}{\left[F_{0}\left(\ell_{3}^{\left(M=0\right)}\right)-F_{0}\left(\ell_{4}^{\left(M=0\right)}\right)-F_{0}\left(L-\ell_{3}^{\left(M=0\right)}\right)+F_{0}\left(L-\ell_{4}^{\left(M=0\right)}\right)\right]}^{2},$$

and that of the mean force is

(46) $$\sigma_{f_{m}}^{2}=\frac{\sigma_{W}^{2}}{{\left(\ell_{4}^{\left(M=0\right)}-\ell_{3}^{\left(M=0\right)}\right)}^{2}}.$$

By splitting the work in *N* cycles, the output work variability can be reduced by the standard $N^{-1/2}$ factor. Moreover, this will also divide the $\ell_{4}-\ell_{2}$ interval, which will also result in a more uniform force.

It could be argued that there is no need for the *M* and *I* bits to be quantum. We next discuss our choice. The fundamental laws of Physics, both classical and quantum, derive from a Hamiltonian formulation. They imply that the evolutions of closed systems fit into the set of canonical or unitary transformations, respectively. Conservation of entropy is built into their structures. Another consequence is that all evolutions are reversible. However, classical and quantum computation models approach these issues in a radically different way. Quantum gates are always assumed to implement unitary transformations. Classical gates, on the other hand, often include non-canonical evolutions, where neither reversibility nor conservation of entropy are observed. This is explained by the fact that the description of irreversible classical gates often neglects interactions that may exchange entropy. When analyzing Szilard Engines that include some information processing, we think that there is an advantage in adopting the quantum information approach. The reason for this choice is that the models of qubit circuits and gates feature explicit conservation of entropy and reversibility. These considerations, together with the intrinsic security that quantum techniques guarantee, justify our preference for quantum over classical information in this particular case.

For the generator mode, the same results apply, considering that it reduces to reversing the motor mode. However, the two operations differ radically in the evolution of the correlation between the *M* and *I* qubits. In the motor mode, it is assumed that at the end of the cycle no correlation is kept between the *M* qubit and any other information stored in the engine. If this were not the case, additional resetting would hamper the work output. In the generator mode, the uncertainty about *M* is reduced after the cycle. Despite the fact that *M* controls the process undergone by the engine, no memory of it should be kept by the end of the cycle.

## 11. Conclusions

As a result of the considerations set forth in the previous section, we claim that it is possible to devise a mechanical system of cylinders that share effective mechanical power through a common informational line, provided that they have access to a thermal reservoir. The potential value of the circulating qubits motivates the study of possible methods to discourage unwanted interceptions. Using a multipartite GHZ state, the information running through the line results completely worthless for any illegitimate user. The procedure described in Section 4 employs CNOT gates both for entangling the qubit before it is sent and for disentangling it upon reception. The qubits that flow between engines are used to unlock thermal energy from the heat reservoir to do mechanical work on the cylinders. They can also be used to store the capacity for work production when heat is supplied to the thermal reservoir at the expense of mechanical work delivered to a rod. The maximum work capacity contained in a single qubit is the well-known Landauer’s energy $k_{B}\left(\ln2\right)T$. When the state is not pure, the value reduces to $k_{B}\left(\ln2\right)T\left(1-S_{i}\right)$, where $S_{i}$ is the entropy of the input qubit. Further, the engine can be tuned to operate between any input ($S_{i}$) and output ($S_{o}$) entropies. The corresponding work has been proved to be $k_{B}\left(\ln2\right)T\left(S_{0}-S_{i}\right)$. Positive and negative values correspond to motor and generator modes, respectively. Within this limit, there is no further bound on the transformation that can be done by a single cylinder on the qubit. Besides, every qubit can be used in sequence by any number of cylinders. This is precisely the requirement that calls for flexibility in the choice of the input and output entropies of the *M* qubits.

When engines are coupled to mechanical systems to do work on them, it is important to select at which force it is delivered. Section 8 describes how the average value of the force exerted on the rod can also be tuned. As it has been shown in Section 10, for any value of the average output work (positive for motor and negative for generator mode), the force has no upper bound. However, there is a lower bound that can only be reduced by either lowering the work per cycle or extending the length *L* of the cylinder.

Accordingly, any force or energy distribution can be implemented and readily changed by just re-tuning the positions at which the walls are introduced or withdrawn. Work can be interchanged between engines at different values of force, as in a generalized lever o hydraulic system. So, power obtained at a weak force can be delivered at any stronger value just by redefining the $\ell_{3}^{\left(M=0,1\right)},\ell_{3}^{\left(M=0,1\right)}$ endpoints. This should be compared to the necessity of new piping or cylinder dimensioning in classical hydraulic circuits.

When temperatures at different ports are not the same, the system follows a Carnot cycle, but, remarkably, the connection between the heat reservoirs is just informational, without any need for pumping fluids through pipes or hoses.

As a final remark, we would like to note that the distribution of mechanical power through communication lines seems to be severely limited by the extremely small value of work per bit that is achieved at usual temperatures, combined with the available transfer rates. However, this restriction might be overcome in the future.

## Appendix A. Energies of a Particle in A Cylindrical Compartment

The energy spectrum for a particle of mass *m* in a cylindrical box of length *ℓ* and radius *R* is given by the succession [68]:

(A1) $$E_{n_{1},n_{2},n_{3}}=\frac{\hbar^{2}}{2m}\;\left[{\left(\frac{\gamma_{n_{2},n_{3}}}{R}\right)}^{2}+{\left(\frac{n_{1}\pi }{\ell }\right)}^{2}\right],$$

where $\gamma_{n_{2},n_{3}}$ is the $n_{2}$-th root of the $n_{3}$-th Bessel function of the first kind, and $n_{1},n_{2},n_{3}$ are positive integers. The partition function is

(A2) $$Z_{0}\left(\ell \right)=\sum_{n_{1},n_{2},n_{3}}\exp\left(-\beta E_{n_{1},n_{2},n_{3}}\right),$$

which factorizes into $Z_{\rho }\left(R\right)Z_{z}\left(\ell \right)$, where

(A3) $$\left\{\begin{array}{ccc} Z_{\rho }\left(R\right) & := & \sum_{n_{2},n_{3}}\exp\left(-\beta \frac{\hbar^{2}}{2m}{\left[\frac{\gamma_{n_{2},n_{3}}}{R}\right]}^{2}\right)\\ Z_{z}\left(\ell \right) & := & \;\;\sum_{n_{1}}\exp\left(-\beta \frac{\hbar^{2}}{2m}{\left[\frac{n_{1}\pi }{\ell }\right]}^{2}\right) \end{array}\right..$$

The Free Energy is

(A4) $$F_{0}\left(\ell \right)=-\frac{1}{\beta }\ln Z_{0}\left(\ell \right).$$

Note that $F_{0}\left(\ell \right)$ is divergent as ℓ→0.

## Appendix B. Location Qubit

In this Appendix we justify the natural emergence of a left-right location or internal qubit, represented by *I*, following the wall insertion at a one-particle Szilard cylinder. Neglecting any tunneling, the Hamiltonian commutes with the left–right projection and the energy spectrum for the left–right subspaces can be obtained from Equation (A1). The Hilbert space $H_{P}$ of the particle can be described as the direct sum of two orthogonal subspaces $H_{P}=H_{A}⊕H_{B}$. They contain the states with the particle on the left or the right side, respectively. Their particular Hamiltonians are ${\hat{H}}_{A}\left(\ell \right)$ and ${\hat{H}}_{B}\left(\ell \right)$. According to Appendix A, their eigenvalues are given by successions

(A5) $$\begin{array}{cc} E_{n_{1},n_{2},n_{3}}^{\left(A\right)}= & \frac{\hbar^{2}}{2m}\;\left[{\left(\frac{\gamma_{n_{2},n_{3}}}{R}\right)}^{2}+{\left(\frac{n_{1}\pi }{\ell }\right)}^{2}\right]\\ E_{n_{1},n_{2},n_{3}}^{\left(B\right)}= & \frac{\hbar^{2}}{2m}\;\left[{\left(\frac{\gamma_{n_{2},n_{3}}}{R}\right)}^{2}+{\left(\frac{n_{1}\pi }{L-\ell }\right)}^{2}\right]. \end{array}$$

Disregarding the spin, the eigenvectors of the Hamiltonians form a basis for each subspace. Therefore, the space $H_{P}$ is spanned by the set of vectors $|I,n_{1},n_{2},n_{3}\rangle$, where $n_{1},n_{2},n_{3}$ are positive integers and *I* is either 0 or 1, representing whether the particle in on the left or the right, respectively. Accordingly, the Hilbert space $H_{P}=H_{I}⊗H_{T}$ factorizes into a two-dimensional $H_{I}$ and an enumerable infinite dimensional space $H_{T}$. The first one contains a qubit, that will be referred to as *I*, while the second one is spanned by the vectors identified by the triplet $n_{1},n_{2},n_{3}$ and represents a quantum system T. In this work, as well as in Reference [1], the system T is always assumed to be in thermal equilibrium. The state of the system can always be expressed by

(A6) $$\rho =p_{0}|0\rangle \langle 0|⊗\frac{e^{-\beta {\hat{H}}_{A}\left(\ell \right)}}{Z_{A}\left(\ell \right)}+\left(1-p_{0}\right)|1\rangle \langle 1|⊗\frac{e^{-\beta {\hat{H}}_{B}\left(\ell \right)}}{Z_{B}\left(\ell \right)},$$

where $\beta ={\left(k_{B}T\right)}^{-1}$, $Z_{A}\left(\ell \right),Z_{B}\left(\ell \right)$ are the partition functions of Hamiltonians ${\hat{H}}_{A}\left(\ell \right),{\hat{H}}_{B}\left(\ell \right)$, respectively, and $p_{1}$ is the marginal probability of finding the particle on the left side.

## Appendix C. Employment of A CNOT Gate to Locate the Particle

In this appendix we describe the role played by the CNOT gate in feed-back control at Quantum Szilard Engines. We show how it can be employed to model the measurement stages, without introducing spurious entropy contributions.

Within the context of Information Engines, it is necessary to account carefully for the entropy changes in the different parts of a system. Unitary gates are entropy neutral. In qubit systems with degenerate Hamiltonians, it is safe to say that they provide operations that neither contribute nor subtract resources. For this reason, we will use the binary CNOT gate and apply it to model the measurement stages.

In order to clarify the entropy interchange, let us further assume that a qubit *M* is fed in a pure $|0\rangle$ state (a more general case is considered in Section 6). It is intended to host the result of measuring the *I* qubit, whose initial state is

(A7) $$\rho_{\mathrm{before}}^{\left(I\right)}=p_{0}|0\rangle \langle 0|+\left(1-p_{0}\right)|1\rangle \langle 1|.$$

After a CNOT, controlled by *I* and targeted at *M*, the resulting (IM) joint state is given by

(A8) $$\rho_{\mathrm{after}}^{\left(IM\right)}=p_{0}|00\rangle \langle 00|+\left(1-p_{0}\right)|11\rangle 11$$

Now *M* can be used to select what process is to be launched. The choice is aimed at driving the *I* qubit to a given target state. Once it is reached, the uncertainty about *I* has been transfered to *M*. Entropy in the joint (IM) system has been conserved.

According to the preceding considerations, the measurement followed by a conditioned action can be adequately represented by a CNOT gate, supplied with an extra qubit (i.e., the *M* qubit) and a posterior evolution controlled by *M*. After the process, there is a transfer of entropy to *M*.
